# What is quality in assisted living technology? The ARCHIE framework for effective telehealth and telecare services

**DOI:** 10.1186/s12916-015-0279-6

**Published:** 2015-04-23

**Authors:** Trisha Greenhalgh, Rob Procter, Joe Wherton, Paul Sugarhood, Sue Hinder, Mark Rouncefield

**Affiliations:** Department of Primary Care Health Sciences, University of Oxford, 2nd floor, New Radcliffe House, Walton St, Oxford, OX2 6GG UK; Department of Computer Science, Queen Mary University, Coventry, UK; Centre for Primary Care and Public Health, Barts and the London School of Medicine and Dentistry, London, UK; East London NHS Foundation Trust, London, UK; Department of Computing, Lancaster University, Lancaster, UK

**Keywords:** Telehealth, Telecare, Multi-morbidity, Quality, Co-design, Ethnography

## Abstract

**Background:**

We sought to define quality in telehealth and telecare with the aim of improving the proportion of patients who receive appropriate, acceptable and workable technologies and services to support them living with illness or disability.

**Methods:**

This was a three-phase study: (1) interviews with seven technology suppliers and 14 service providers, (2) ethnographic case studies of 40 people, 60 to 98 years old, with multi-morbidity and assisted living needs and (3) 10 co-design workshops. In phase 1, we explored barriers to uptake of telehealth and telecare. In phase 2, we used ethnographic methods to build a detailed picture of participants’ lives, illness experiences and technology use. In phase 3, we brought users and their carers together with suppliers and providers to derive quality principles for assistive technology products and services.

**Results:**

Interviews identified practical, material and organisational barriers to smooth introduction and continued support of assistive technologies. The experience of multi-morbidity was characterised by multiple, mutually reinforcing and inexorably worsening impairments, producing diverse and unique care challenges. Participants and their carers managed these pragmatically, obtaining technologies and adapting the home. Installed technologies were rarely fit for purpose. Support services for technologies made high (and sometimes oppressive) demands on users. Six principles emerged from the workshops. Quality telehealth or telecare is 1) ANCHORED in a shared understanding of what matters to the user; 2) REALISTIC about the natural history of illness; 3) CO-CREATIVE, evolving and adapting solutions with users; 4) HUMAN, supported through interpersonal relationships and social networks; 5) INTEGRATED, through attention to mutual awareness and knowledge sharing; 6) EVALUATED to drive system learning.

**Conclusions:**

Technological advances are important, but must be underpinned by industry and service providers following a user-centred approach to design and delivery. For the ARCHIE principles to be realised, the sector requires: (1) a shift in *focus* from product (‘assistive technologies’) to performance (‘supporting technologies-in-use’); (2) a shift in the *commissioning model* from standardised to personalised home care contracts; and (3) a shift in the *design model* from ‘walled garden’, branded products to inter-operable components that can be combined and used flexibly across devices and platforms.

Please see related article: http://dx.doi.org/10.1186/s12916-015-0305-8.

## Background

### Assisted living technologies

Assisted living (or ‘assistive’) technologies include telehealth (remote monitoring for clinical biomarkers) and telecare (for example, alarms, sensors, reminders), designed to deliver health and social care services to the home [1]. The arguments for developing them are well rehearsed [2-5], though not unchallenged [3,6-8]. As society ages (so the argument goes), more and more people have chronic conditions; assistive technologies, alongside self-management by patient and carer, will help monitor, treat and even prevent such conditions, thereby improving quality and length of life while also relieving pressure on increasingly stretched health and social care services.

Driven partly by concerns about costs of long-term care, investment in assistive technologies from industry, government and research sponsors is high. In the UK, for example, the Department of Health’s £31 M Whole Systems Demonstrator trial (2008 to 2011) was, as its name implies, designed to *demonstrate* the effectiveness and cost effectiveness of ‘whole system’ approaches to technology introduction and use [9] – though arguably, it generated more controversies than it resolved [10]. The Technology Strategy Board (TSB) allocated £25 M to its Assisted Living Innovation Platform (ALIP) in 2008 to 2011, with many projects attracting matched industry funding. ALIP’s main sequel, DALLAS (Delivering Assisted Living Lifestyles at Scale), a £23 M partnership between TSB, the National Institute for Health Research (NIHR) and government, runs from 2011 to 2015. The European FP7 (2008 to 2013) and Horizon 2020 initiatives include large, inter-sectoral programmes to produce assistive technologies at scale and drive these into production and widespread use [3].

We have previously demonstrated, through discourse analysis of academic, policy and lay texts, that such programmes are predicated on a modernist vision of the ‘smart’ home in which ubiquitous technologies, seamlessly integrated with health and social care information systems, will enable dignified ageing by preventing, minimising or compensating for the effects of degenerative disease [3]. The assumption behind this vision is that assistive technologies, if optimally developed and implemented at scale by a thriving and innovation-driven technology industry, will generate social change and thereby (at least partly) solve the uncomfortable problem of what we should do with the growing ‘burden’ of ageing and dependent citizens – while also saving money.

But this vision of a technology-supported ‘better society’ remains elusive, for two reasons. First, there is persistent over-confidence in the capacity of technological innovations to configure the future. Second, there are material and ethical questions of how chronic illness and suffering affect people’s capacity to live in the world. These themes have been addressed in what might be called the ‘critical’ literature on assistive technologies, summarised briefly below.

### The myth of the smart home

In *Designing a Digital Future*, Dourish and Bell expose the myth (now 25 years old) of the ‘smart home’ [11]. They highlight designers’ preoccupation with the *proximate future,* an imminent era of ‘calm’ – ever-present, invisible, reliable – technology, when the mess and hassle (for example, glitches, bugs, interoperability, intellectual property, information governance, set-up costs and so on) associated with technologies have been resolved, leading to *‘a future saturated with technology’* (page 22). This utopian future, they warn, will never materialise and needs to be purged from our dreams and plans:

*‘Lift the cover, peer behind the panels, or look underneath the floor, and you will find a maze of cables, connectors, and infrastructural components…. Push further, and you will encounter regulatory authorities who authorize interventions and certify qualified individuals, committees that resolve conflicting demands in the process of setting standards, governments that set policy, bureaucrats who implement it, marketers who shape our views of the role of the infrastructure in our lives, and more. Mess is always nearby’* (page 4) [11].

In the modernist dreams of policymakers and designers, a new generation of more sophisticated information and communication technologies (ICTs) is what is needed to rescue people from this ‘mess’ and to bring order to their affairs [3]. Yet the reality often proves otherwise, especially in applications where a close fit between technology and user requirements is essential, but the heterogeneity of the latter – and, in some circles, a privileging of quantitative research over qualitative – limits our understanding of them [12]. Examining programmes through a socio-technical lens, it is likely that successful technology development, installation and use will be challenged by contestation about standards (clinical and technical), policies (national and local), practicalities of use (the ubiquitous model-reality gap in all its forms), service support (what is the role of the physician, specialist nurse, call centre or data processing hub in the ongoing support of an installed device?) and commercial interests (including manufacturers’ profit motive and associated barriers to interoperability). Rather than being ‘plug and play’, assistive technologies will always need skilled human work, inter-sectoral negotiation and a *social* infrastructure to ensure that they ‘work’.

### The uniqueness of assisted living needs

Modernist research on assistive technologies, led by computer scientists, is remarkably thin on clinical detail. Because of this, it tends to generate superficially plausible solutions that may prove unusable in practice because their design fails to take account of how multiple medical conditions affect a person’s ability to understand and operate a technical device – or of variation in how people may want to use the device and, indeed, what they may want to use it *for* [3,12-15]. Yet clinical experience readily demonstrates that people’s illnesses and impairments are unique, and every individual will have different goals and a different view of how technologies will best help them.

Everyday ethics is a key theme in critical assistive technology research. As Heidegger showed, we use technologies (when we can, and to the extent that they ‘work’) to do things and make things – and at a more abstract level, to *achieve what matters to us* [13]. When a technology interferes with what matters to people (for example, when it makes the bedroom look and feel like a hospital ward), they quickly reject it.

Underpinned by this critical perspective, and with the aim of improving the proportion of patients who receive appropriate, acceptable and workable technologies and services to support them living with illness or disability, we sought to answer the following research question: what is quality in the design, implementation and use of telehealth and telecare, and how might we achieve such quality?

## Methods

### The ATHENE study

The ATHENE study (Assistive Technologies for Healthy Living in Elders: Needs Assessment by Ethnography), which ran from 2010 to 2013, was funded by the Technology Strategy Board Assisted Living Innovation Platform programme. It sought to produce a rich understanding of the lived experiences and needs of older people with multi-morbidity and to explore how those involved in providing and supporting the technology – technology suppliers, health and social care providers – can work with care recipients and carers to ‘co-produce’ technologies and service solutions. The ATHENE study had an external steering group with an independent lay chair and representatives from academia, policy, health and social care providers, technology suppliers and service users (including technology users). Ethical approval was gained from Queen Mary University of London Research Ethics Committee (QMREC2011/38 1 June 2011), Harrow NHS Research Ethics Committee (11/LO/0737, 8 July 2011) and subsequent amendments.

The study consisted of three phases. Phase 1 involved initial interviews with 21 key stakeholders from technology suppliers (n = 7) and service provider organisations (n = 14). Phase 2 consisted of detailed ethnographic studies of 40 individual cases, conducted in and around the person’s home to map the complex healthcare, social care and socio-cultural needs of older people and their carers, encompassing a range of ethnic and social groups. Phase 3 took forward exemplar cases and used participatory design methods to explore how older people and their families might work directly with industry designers and service providers to produce fit-for-purpose technologies (either new or adapted) along with appropriate service support, that fit in with people’s care needs and lifestyles. Previous papers from the ATHENE project have reported the methodology [16], findings from interviews with suppliers and service providers theorised using diffusion of innovations theory [17], and analyses of the ethnographic data from a sociological perspective [13] and from a computer-supported cooperative work perspective [18].

### Sample and setting

The study was undertaken across two sites, in London and Manchester, both characterised by ethnic and socio-economic diversity with a predominance of poverty and deprivation. The sample of seven technology suppliers was drawn from a range of medium and large companies that made and supported assistive technologies. They were recruited via networking events on assisted living and/or industry representatives on our steering group; we set no restrictions on the particular technologies they made. We have deliberately not given detailed information about these organisations to preserve anonymity. The 14 service provider representatives were drawn from 10 telecare and telehealth support services, comprising six local authorities, one private, two NHS trusts and one voluntary sector. Demographic characteristics of the participants living with multi-morbidity are summarised in Table 1 and medical conditions (objective and subjective) in Table 2.

**Table 1:** **Summary of participants in phase 2**

**Age (median, range) 81 (60 to 98) years**	**Number**
Gender	
Male	13
Female	27
Ethnicity	
White British	24
Other European	1
South Asian	4
Chinese	3
Caribbean	5
African	2
Housing status	
Own house or flat	19
Privately rented	1
Housing association	7
Local authority	10
Sheltered housing (that is, with resident warden)	3
Living arrangements	
Alone	18
With partner only	13
With partner and/or other carer	9

**Table 2:** **Summary of medical conditions and subjective impairments in phase 2 participants**

**Objective medical conditions**	**Number**
Neurological conditions (stroke, Parkinson’s, other tremor, severe migraine, past polio, not formally diagnosed)	20
Arthritis	14
High blood pressure and/or high cholesterol	14
Chronic respiratory disease (asthma, chronic obstructive pulmonary disease)	13
Diabetes	11
Macular degeneration, glaucoma or cataract	11
Coronary heart disease	10
Depression, anxiety or psychological stress	7
Dementia, cognitive or memory problems	7
Side effects from medication	7
Trauma (for example, recent or persisting effect of past fracture)	6
Swollen feet without formal diagnosis	3
Cancer	2
Other (e.g. urogenital, kidney failure, anaemia, tendency to infections, hormone deficiency, peptic ulcer, sleep apnoea, deafness)	16
**Subjective impairments affecting basic day to day tasks**	
Generalised tiredness/low energy	23
Significant and persistent pain	18
Stiffness or weakness in joints and/or muscles	18
Shortness of breath	13
Poor or no vision	11
Unsteadiness, dizziness or balance problems	9
Poor cognitive capacity, concentration or confidence	11
One or more limbs paralysed	7
Bulky device affecting mobility (e.g. oxygen cylinder, catheter)	7
Incontinence	6
Difficulty with fine finger movements and/or writing	5
Blackouts, loss of consciousness or perceived risk of these	5
Physical bulk (obesity, severely swollen legs)	4
Wandering	2

### Theoretical position

Our critical (in the sense of ‘critique’, not ‘criticism’) perspective on assistive technologies rejects the technological determinism and naïve utopianism of many studies of telehealth and telecare [3]. It is grounded in phenomenological philosophy, especially Merleau-Ponty’s work on perception and Heidegger’s concept of how technology, when ‘ready-to-hand’ (that is, smoothly aligned with a person’s bodily and mental functions), extends both sensory perception (the capacity to feel, see, hear and so on) and motor intentionality (the capacity to act purposefully using the body) [19,20]. Within this framing, we align with others’ research on the sociology of the body (particularly Pickard and Rogers on the lived experience of illness and ageing [21,22]); the ‘moral turn’ in the social sciences, particularly Sayer’s notion of ‘what matters to people’ [23] and Mort *et al*.’s work on the social and ethical implications of telecare technologies, which, in order to ‘work’, must be nested in networks of accountable human relations and responsibilities [12].

Our work also aligns with other research in the critical ethnography tradition, including what Star has called ‘the ethnography of [technological] infrastructure’ [24,25]; ‘health and place’ geography, in which healthcare technologies and their use are considered in the context of the physical, material and symbolic spaces of the home and community, and the networks of family and social relations linked to these [26,27]; and critical nursing studies, in which the old-fashioned dualism ‘high-tech’ versus ‘high-touch’ is replaced with a more contemporary theorisation of how technology can *support* intimate nursing care of the body [15]. Much of this critical literature comes from the Netherlands, Sweden and Norway, where the study of technology-in-use (performative, practice-focused and using ethnography in real-world settings) has high credibility. But such approaches currently have less of a foothold in the UK and North America, where research funders have tended to privilege development of advanced technology solutions and randomised trials to ‘demonstrate’ these [9].

A phenomenological lens begins with the intended technology user’s perceptions and desires and asks how technology could augment the former and help achieve the latter. This approach thus has a very different starting-point from studies emphasising how assistive technologies could be used for supporting the biomedical agenda (for example, monitoring of disease). In the past, designers assumed that computers would be used in homes for the same tasks as they were used for in offices (for example, filing, calculating). Early computers aimed at the home market emphasised how important these tasks were (or were likely to become) in the modern home. It was only when people began to envisage home computers in a radically different way, to ‘digitally enable’ the *home activities that mattered to people* like playing games and listening to music, that home computing took off at scale [11]. In the study reported here, we sought to apply this general principle to assistive technologies. Starting from the premise that attempts to turn the home into a mini-hospital are doomed to failure, we developed and refined ethnographic techniques to build a rich picture of *how people actually live* with multi-morbidity. We focused in particular on the experience of illness and suffering and how people use (or why they choose not to use) particular technologies.

Phenomenology underpins the science of experience-based design, which takes the patient’s ‘ordinary experience’ as the starting point for clinical microsystem and wider health system redesign [28]. As noted above, few technologies designed for the so-called smart home are ‘plug and play’; there is an emerging literature on how individuals adapt and customise them to fit with personal needs and capabilities and with the material constraints of their local setting [11].

### Phase 1: Interviews

Sixteen semi-structured interviews were conducted with a purposeful sample of 21 participants (7 technology designers and 14 service providers) involved in the development and provision of telecare in the UK; full details are given in a separate paper [17]. Questions focused on perceived challenges to the uptake and use of telecare; the technology design process; the installation and support of telecare technologies; and views about future developments. The interview protocol was adapted as the study progressed to explore emerging themes in more detail. Interviews were recorded with consent and transcribed.

### Phase 2: Ethnographic studies

In the ethnographic studies (described in detail elsewhere [13]), we visited 40 participants at home, each on several occasions one to two weeks apart, and encouraged them to help us build a rich picture of their lives, including their daily activities and what mattered to them. Our techniques include cultural probes (in which participants become co-ethnographers, using cameras, diaries and scrapbooks to collect data about their lives); home tours (in which the participant takes the researcher round their home, describing each room’s significance and activities that occur in it) and narrative interviews (in which a conversational format is used to explore stories about the person’s life raised by them) [16,29]. We analysed the multi-modal dataset thematically and applied narrative as a summarising and synthesizing device to produce rich individual case studies (four to ten pages long) of each participant, presented in a semi-structured format that covered: the social and cultural and historical context; the participant’s experience of ageing and ill health; key people (lay and professional) in their life; what mattered to them; technologies in their home and life; key material properties (of the technologies) and key capabilities (of the individual to operate and interpret these technologies); and specific incidents of using (or choosing not to use) technologies.

### Phase 3: Co-design workshops

Ten workshops were conducted with 61 participants. Four were held with a total of 30 end-users (case participants, their carers and third-sector advocates); three were held with a total of 18 service provider representatives (occupational therapists, nurses, monitoring operators, technicians, service managers, commissioners); and two were held with 13 technology industry representatives (designers, engineers, business development, marketing). The final workshop brought together 11 representatives from across these different user and stakeholder groups.

Co-design workshops are an established participatory design approach to help users and stakeholders articulate existing practices, identify challenges and develop new ideas [30]. They have much affinity with Robert and colleagues’ work on experience-based co-design of clinical services [31], but include a more explicit focus on the design, adaptation and use of technologies. During the workshops, we presented vignettes from the ethnographic work to communicate the lived experience of older users, promote the sharing of personal stories and elicit ideas about how the technologies and services currently on offer could be improved.

**Figure 1:**
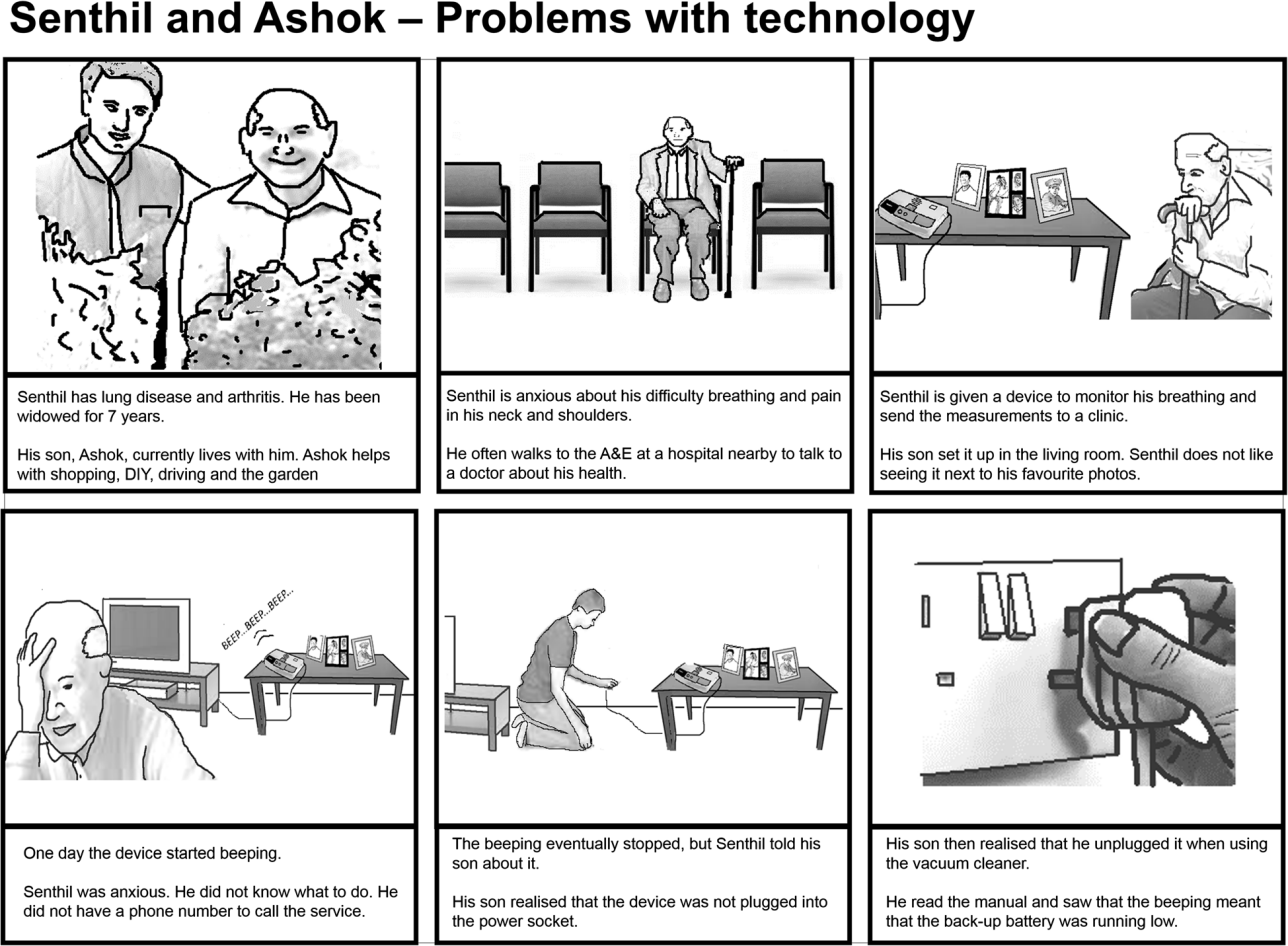
**Example of ‘cartoon strip’ approach to generating discussion about case scenarios.** In this example, Frame 1 introduces the characters (Senthil and his son, Ashok). Frame 2 describes Ashok’s health problems and frequent visits to the clinic. Frame 3 describes the installation of a telehealth device to monitor Santhil’s blood pressure and oxygen saturation. In Frame 4, Senthil is confused and concerned about a beeping sound from the device. In Frame 5, Ashok later realises that the device is not plugged into the power socket, and that the beeping indicates low battery. In Frame 6, Ashok plugs the device back into the power socket and reminds Senthil not to remove it.

In the four technology user workshops (users, carers and advocates), vignettes were presented using a ‘storyboard’ format, which depicted, in cartoon-strip format, a narrative in a series of frames (see example in Figure 1). The stories were fictional but based on real accounts from the ethnography of problems encountered with assistive technologies. Workshop participants considered the material features of technologies, facilitated by a sorting exercise of cards depicting specific devices’ design features. They also considered aspects of telehealth and telecare service provision, facilitated using a flow diagram of the assistive technology provision process – ‘assessment’, ‘decision for telecare/telehealth’, ‘installation and training’, and ‘review’.

The four service provider and two technology industry workshops began with a presentation of an anonymised case study from phase 2 along with additional data extracts (stories, quotes, probe materials and photographs). Participants were sent the example case summary prior to each workshop (with the index participant’s consent) and asked to reflect on three questions: (1) Bearing in mind what matters to this person, how could their life be improved through a technology or service?; (2) What would be the issues/challenges in implementing one of these solutions and how might these be overcome?; and (3) How might the technology or service be sustained and adapted over time? Following discussions on the vignettes, participants considered the implications for service design, facilitated by a flow-diagram of the service delivery process, such as ‘assessment’ and ‘review’. The industry workshop centred on implications for technology development, articulated in design terminology, such as ‘requirements gathering’, ‘prototyping’, ‘field trials’, ‘user feedback’.

All workshops were audio recorded and professionally transcribed. The end-user, industry and service provider workshops were analysed separately using a constant comparative approach [32] to summarise participants’ perspectives on the ethnographic data and priorities for technology and service improvements. Common themes across the first nine workshops were synthesised to sharpen the focus for the final cross-sector workshop, which brought together the different user and stakeholder groups. An anonymised case summary was used to provoke discussion on how collaboration between formal and informal carers could be supported through technical and social systems. Prompt cards were used for participants to brainstorm about whom they would like to communicate with across the care network, and what type of information would help them support the technology user more effectively. Again, this workshop was recorded and transcribed.

### Data analysis

We used the 21 transcribed stakeholder interviews from phase 1, 40 individual case studies from phase 2, and the written summaries of the 10 co-design workshops from phase 3 as an intermediate dataset. JW and TG re-analysed these texts thematically using the question ‘what is quality in telehealth/telecare provision?’ as a guiding question. Each researcher independently looked through the texts and identified characteristics of ‘quality’ telehealth or telecare and also examples of (real or perceived) quality failures. These were shared among the wider research team and refined by discussion.

## Results

### Stakeholder interviews

Participants identified multiple interacting influences on the adoption, assimilation, implementation and sustainability of telecare. This included attributes of the technology (for example, relative advantage over existing arrangements, low complexity, risk involved in adoption), characteristics of the intended user (especially their physical and cognitive capabilities); the extent and nature of social influence (for example, limited awareness of these technologies among many health professionals, who, therefore, did not mention them to their patients); low levels of organisational innovativeness (due partly but not entirely to squeezed budgets); low levels of organisational readiness for telecare technologies (due partly to a perception that these innovations would not be cost-effective); weaknesses in the assimilation process (especially inadequate assessment and tailoring to the individual and care network); weak embedding of telecare in the business-as-usual of the various organisations who might contribute to the support network; and poor links between users and developers at the design stage [17].

Findings from this phase highlighted that solutions for assisted living are complex innovations requiring input from, and coordination between, people and organisations. To promote adoption and use, the different contextual factors must be specified, understood and addressed. A number of important questions were raised that sat largely outside the domain of technology design, particularly with regard to how we optimise the process of assessment and personal ‘tailoring’ of an off-the-shelf device: how to overcome organisational inertia and lack of resource when introducing assistive technology services; how to make the services more cost-effective (and, hence, more attractive to commissioners and purchasers); and how to optimise the long-term support for the technology user so that it is sustained as a ‘working’ technology in the long term. Full details of the findings from this phase are presented elsewhere [17].

### Ethnographic case studies

Our ethnographic research revealed a huge diversity of individual and family circumstances, medical conditions (see Table 2), personal priorities, physical and cognitive capabilities, installed technologies, home environments, and extent of support from family and friends. The following anonymised excerpt from our case summaries illustrates the uniqueness of assisted living needs in people with multi-morbidity while also highlighting a number of system-level problems that were common across many assistive technology users (and non-users) in both study sites:

*Walter, a white British man aged 72, is single and lives with Christine [friend] along with her partner Phil, and their four children. […] He has chronic obstructive pulmonary disease (COPD) as a result, he thinks, of his lifelong smoking. He says he used to have home oxygen last year for the COPD but the nurse told him he didn’t need it because his oxygen levels were OK. Walter disagrees with the nurse’s assessment. He said he feels he needs oxygen sometimes. He has high blood pressure, and also prostate problems that have led to urinary incontinence, for which he wears pads. Walter says he can go [to the pub] for a couple of pints, go to the toilet, and then still find himself ‘leaking’. Because of his urinary problems and his breathing, Walter’s sleep is very disturbed.*

*While Walter says, ‘I don’t have any problems, memory wise’, Christine explains that he does have memory problems (for example, he naps for an hour then wakes up and thinks it is the next morning), and he also has vacant periods for reasons that are unclear. He is waiting for an appointment for a brain scan. At night, Walter sometimes wanders about the house and tries to cook. This is not safe (he has burned toast in the past), so Christine has put a lock on the kitchen door.*

*Walter spends most of his time indoors watching TV, going outside periodically for a cigarette. The house is small and very cluttered with a tiny outside yard, and with 7 people and numerous animals, there is not much spare space. The nurses have talked about Walter having a wheelchair but Christine is not keen. There is no room in the cramped house to store it. Walter does not feel he needs a wheelchair. What they would really like is for the council to put a large gate on the back garden fence so they can get the car in, making it easier to get Walter into the car, especially on cold and rainy days.*

*Walter is on various medications, tablets and inhalers for the COPD and more tablets for the prostate problem. He has regular visits from health professionals and is somewhat confused about these. He has had telehealth equipment installed for about a year but he does not use it now and nobody has been to collect it […]. The devices include an oximeter, blood pressure monitor, thermometer and weighing scales. He still has a nebuliser, which he uses occasionally.*

*Walter says that someone talked about him having a pendant alarm but it didn’t arrive. He had fallen 3 or 4 times in his bedroom and he didn’t know what had caused the falls. He would very much like to have a pendant alarm.*

*A major practical issue for Walter and his adoptive family is his incontinence. Christine says that the incontinence pads cost £13 per pack and Walter sometimes goes through 2 packs per week. This is a significant drain on the family finances. They tried unsuccessfully to get them on prescription, and are now trying again. Christine explains:*

*‘Last time, the nurse said, “Write down what he drinks, how often he drinks it, how many times he goes for a wee, see if you can measure his wee”, and I’m at it ‘You’re joking aren’t you, I’ve got four kids in the house, I can’t be running in and out of the toilet when he’s peeing’. Then when I wrote it all down she said “no, not good enough, he’s not entitled to them.” It got to the point where he was leaking on to the couch and they’re telling me he’s not good enough, you know what I mean?’*

*Walter gave up using the telehealth equipment because the times of taking the measurements were not convenient for his family. Walter needed Christine to help him take the measurements, but because of his sleep disturbance, he did not get up until late morning – by which time Christine was up and out of the house. They only used the equipment about six or eight times in total. The researcher asks whether the telehealth equipment was useful while he was using it. He replies there were lots of health professionals coming and going. He thought the point of the telehealth equipment was to save health professionals time but, he says, it didn’t seem to.*

Like many other cases in our sample, Walter has multiple medical problems that interact to produce the combination of low energy, low motivation and limited physical and cognitive capacity. Some of the installed technologies (for example, the weighing scales and thermometer) do not seem to match his medical conditions. Importantly, the very conditions for which he was deemed to need assistive technologies make him incapable of using them, mainly because of their *non-specific* effects on his energy and motivation. His medical conditions are neither stable nor fully diagnosed (his vacant periods, for example, may or may not have a neurological origin). What matters to him is getting out of the house – either to the back yard for a cigarette or to the pub for his pint.

Walter’s case illustrates that telehealth relies on the person’s own ability to use the equipment and/or on the ability and willingness of their family and friends to help them do so. Far from being ‘plug and play’, allowing remote monitoring of the individual whatever their capability and motivation, the technology makes high demands for cooperation and conformity – for example, in this telehealth service, a phone call must be made at 10 am. If the family routine does not mesh with that of the telehealth service, the technology quickly falls into disuse.

It is ironic that Walter has been equipped with a telehealth package costing several hundred pounds (which he cannot use because the routine for sending the readings does not align with the wider routine of his host family), but has been classified as ineligible for the simpler and cheaper solution of incontinence pads on prescription. The formalised assessment process, involving ‘objective’ measurement of the amount of urine passed with a view to categorising Walter as either ‘needing’ or ‘not needing’ incontinence pads, contrasts with Christine’s account from a carer’s perspective: she knows Walter intimately, and can describe from a subjective, lived-body perspective how the incontinence affects him and the rest of the family. Similarly, the privileging of ‘objective’ measures of oxygen ‘need’ led to Walter’s oxygen service being withdrawn even though it gave him subjective benefit. The cases study thus raises (but does not answer) the question of whose perspective should ‘count’ in the provision of telehealth and other technologies from a limited budget.

Walter’s case illustrates a much wider finding of our ethnographic work – that off-the-shelf technologies were rarely useful or usable by people with complex medical needs. Rather, successful solutions, where they occurred, had been produced for the participant through ‘bricolage’ – pragmatic, needs-focused customisation of the technology by a person who knew them well [18]. But his case also illustrates the limitations of bricolage, given the current technological and service climate: Christine and Phil have made numerous material adaptations to the house (for example, the kitchen door lock) to make it safe and accessible for Walter, but because the supply of telemonitoring service and domiciliary oxygen are driven by system-level protocols, criteria and standards, Walter’s carers are powerless to customise these to meet his needs.

We have described Walter’s case in depth to highlight key themes that were evident across many cases in our sample. Much more rarely, technologies were helpful and valued – and this occurred when they extended existing support from either family or professional carers. For example, participant Bonnie (aged 81) also suffered from chronic obstructive pulmonary disease; her daughter Carol liked the telehealth equipment, mainly because the oximeter readings often allowed her to convince her mother that there was no need to panic. However, using the equipment to obtain these reassuring readings involved Carol making face-to-face visits and doing considerable additional work. In this respect, the technology was neither labour saving nor time saving.

In sum, this ethnographic work revealed how people’s capacities and capabilities, shaped by both socio-cultural frames and the physical and cognitive effects of illness and ageing, align to a greater or lesser extent with the material and symbolic properties of technologies in particular settings. Most crucially, these rich case studies have begun to characterise, in close clinical detail, how multi-morbidity affects people’s ability to use technologies and the (often limited) extent to which technologies can prevent or attenuate the suffering of multi-morbidity. Further examples from our 40 ethnographic case studies are given in other academic publications [13,18]. In addition, 23 participants consented for the full text of their case summary to be published on the open-access ATHENE website [33].

### Co-design workshops

Workshops provided a lively and creative forum for people with assisted living needs, informal carers, service providers and technology suppliers to discuss the ethnographic data, share their experiences, and elicit technology and service design ideas to address issues raised. Key themes relating to the design of quality telehealth and telecare solutions included customisation and adaptation; information sharing and coordination; and ongoing social interaction and support. These are presented in turn below.

#### Customisation and adaptation

Users, technology designers and service providers repeatedly emphasised the need to provide tailored solutions and gave numerous examples of barriers to effective customisation. The initial assessment visit was considered particularly critical to getting to know the patient/client, especially the specifics of how they live and their experience of their health condition. An important component of this visit must be to spend time talking with the end user and those close to them in order to find out what matters to them and ensure that any technology solutions are fully personalised. Workshop participants commented that this counsel of perfection was difficult to achieve in current clinical and social care practice, and that few technology suppliers are sufficiently skilled to undertake this work (which was considered to require clinical or clinically-related training).

*‘However much training you do and however good people are at delivering telecare, unless they take into account the person’s situation and how they live in their home, it’s going to be rubbish. I mean, ranging from not noticing they’ve got a dog, a large dog, which can muck up the bed sensor something rotten, or, for instance, that they use a wok to cook with, which is not very good if you’ve got a high temperature alarm in the kitchen…But it’s really about talking to the person, spending time with them, not just once. Because the [current] idea is it’s like a prescription, isn’t it? You get this Telecare prescription and ‘there it is, bye’. But actually, it’s something you have got to work with. You’ve got to go back and go back again and make sure you review the need overall…So this always brings it home to me, you know, what you do in your case studies, what really matters…’ (Occupational Therapist)*

Experiences with installing assistive technologies brought participants to the view that practical reasoning is required, focusing on individual contexts, material constraints and the ends that are to be achieved. However, they also felt that the importance of such reasoning had not been fully acknowledged across the services, and that it was difficult to achieve because patients tended to be passed through distinct care teams, each with specific responsibilities and tasks along a so-called care pathway with connotations of an inflexible, ‘production line’ approach.

*‘If you’re working in a service, you want to know, I’m doing this, this, this and if I have done that, then it’s at the end of my duty and I can sort of pass this person on… But in reality, the situations that you face in health and social care, they’re so complex and confused that if you really wanted to address somebody’s needs, it’s like a mini project. Where do you start and where do you end?’ (Occupational Therapist)*

One service provider described a one-off situation in which she was (unusually) able to tailor telecare technology to an individual patient’s needs, because she did not have to follow set procedures and protocols:

‘*Recently I did an OT assessment for a lady who was not eligible for social care. And so I went into – almost like in an advisor capacity, assessed her and everything, but it turned out what she really wanted, what was really of value to her, was completely out of the box, you know. And I kind of made loads of phone calls, I went online, to contact various people and look at websites, as we were doing this… And instead of kind of doing the standard, which I would have normally done, because it was outside of the statutory circuit I could do this. And I sort of felt, you know, this is really quite good, this is much more like a role that I believe would help people. … So it’s not all about the technology itself, it’s also about the approach.’ (Occupational Therapist)*

Beyond the challenge of understanding user requirements, personalisation appeared to be further hindered by the limited range of technologies available locally. Service providers were often ‘locked in’ with particular devices and brands, provided as a standard package, and there were contractual limits on what could be provided by and to whom. Commissioners made the purchasing decisions, and clinicians then had to make the best of what was available. The purchasing model preferred by our participants was characterised by greater engagement between commissioning and service staff; greater control and flexibility to explore and trial different technology options; and a change in relationship between industry and commissioners (which currently assumes purchase of technologies in bulk).

‘*I think some of the problems we’ve got is the equipment in [name of city] was bought by commissioners, non-clinicians, and had there been more engagement with the clinicians who were going to use it, who had an understanding of the patients who were going to use it, it might be slightly different than what we’ve got. They jumped in feet first. … But ultimately, the decision was made on the basis of how much money, how many units, and it was a commissioning decision, not a clinician decision*.’ (Telecare Lead)

#### Information sharing and co-ordination

Participants identified a need to support knowledge sharing and co-ordination within and between care services, as well as across formal and informal care networks. Currently, service chains – with several people involved in supporting an individual patient or client – are complex and lack effective integration and information sharing. To the extent that aspects of the telehealth or telecare service (for example, installation, the monitoring centre) are outsourced to subcontractors, this can add another level of separation.

‘*So if you are referring to another service for some intervention, you could have closed the case hoping the referral you’ve made is then going to do that bit of work. You might have the luxury of actually phoning or contacting that service and discussing something together, but that can be a luxury to be able to do that. Or even if you do that, that service are saying, we’ve got a two month waiting list, we can’t see this person for ages. So again, the barrier, you know, is there physically to actually work together. And then things become a bit sort of strung out, it becomes a bit like Chinese whisper, as things go through different services.*’ (Occupational Therapist)

The workshops identified that improved intra- and inter-agency coordination and information sharing is needed to track users’ changing circumstances and needs, identify any actions that need to be taken in response, and enable continued development and customisation of assistive technology solutions. Participants’ suggestions aligned closely with findings from the computer-supported cooperative work literature that social and technical subsystems should be organised to support collaboration through mutual awareness (the sense of what the other collaborators are doing in order to provide a context for your own activity on a common project) [34] and facilitate sharing of both ‘formal’ knowledge (documented and accessible by people within an organisation) and ‘informal’ knowledge (gained over time through everyday practice, and not generally documented) [35]. Participants suggested, for example, that an assistive technology should be reviewed in conjunction with a routine clinical care visit, which could help sustain engagement with users in a more cost-effective way.

*‘It might be that by knowing that, say, a district nurse is planning a review, we could slot in a couple of simple questions and save the need for another resource, or then catch up with that district nurse afterwards…Or come along, and share that information. I just don’t think we do enough of that and maybe there are some quick wins about when things are planned in.’* (Telehealth Service Manager)

Such collaborative models could potentially overcome many of the difficulties of data integration and patient consent to share personal and health information. But as our workshop discussions highlighted, they presuppose an altruistic and collegial rather than commercial or contractual relationship between different professional staff and their respective organisations. In reality, achieving high motivation across the multiple actors to engage and contribute to the collective task of supporting an individual over time will be hard to achieve. Prioritising the subjective lived experience of the patient over the application of standardised criteria and checklists as the shared quality outcome could go some way to strengthening this collective effort.

*‘I prefer the idea – it’s idealistic maybe, but that everything is looked at because it’s working for the person, it’s the holistic word again, but trying to get that ticking over with everyone understanding what the aims are so we’re all working towards the same thing.’ (Assistive Technology Lead for Adult Care Services, Local Authority)*

Altruism and ‘pro-sociality’ (going beyond formal job requirements and procedures) in healthcare is often seen as an integral part of the job to help the patient. This is because the precise combination and sequence of skills to be used in particular circumstances cannot always be specified in advance [36]. Effective design and delivery of assistive technology therefore relies on spontaneous cooperative acts to deal with task complexity and uncertainty, which in turn requires a collective motivation to address problems as and when they arise. In healthcare settings, frontline staff have first-hand contact with patients, and so they have an advantage in establishing the engagement and empathy that motivates pro-social behaviours. A challenge for telecare and telehealth will be to foster a similar understanding and motivation across all those who contribute to the implementation, maintenance and sustained use of the technology solution.

#### Ongoing social interaction and support

Thirdly, participants talked about the role of social supports to bridge the design-realty gap. Family members usually played a key role in decision making, liaising with the services and solving problems with the technology, stepping in as needed to set up equipment or resolve technical issues. However, human resources within the care network of a patient or client were variable. For users who depended solely on professional services, even minor problems (for example, replacing batteries on a device) could pose significant confusion and disruption:

*‘With the carbon monoxide ones…I didn’t realise it was that, but for months I heard this beep, beep, where the hell is it coming… and it didn’t dawn on me that it was the carbon monoxide one…It wanted a battery, all it needed was a battery. But I didn’t know where the battery was going to go, I couldn’t change it....I put it on the settee behind the cushion…But it took them so long to come and change the battery for me....I rang them and told them. … And it was about two weeks before Christmas that I told them, and then one week passed and they said, “oh he’s off sick”. So it went through Christmas and then New Year and then it was January before they came.’ (Telehealth user Elsie aged 82)*

Participants emphasised that service providers need to assess available social resources, involve them in the design of the care solutions, and be prepared to respond to diverse and often subtle and mundane issues. Service users emphasised a need for staff to reach out to users to create opportunities for interaction (for example, face-to-face or over the phone) to support them with technology, and create a sense of familiarity and ‘being cared for’. Building personal relationships with users and their care networks would, participants said, improve the user’s understanding and perception of the technology, as well as the confidence to raise issues or request further adaptations or support. They felt that investment in these human efforts would greatly increase effectiveness of the technology.

*‘You get, “Oh, you pull this, you pull that,” and you get muddled…We get five minutes, perhaps. They’re used to the piece of equipment, whatever you like to call it. And it is very difficult because, especially in my age group, we look such utter fools in asking for more help to understand what is going on and how it can help. Give us a five-minute talk, you’re lost…And that’s another thing, you see; if you called in perhaps the next day or a couple of days later, and had a cup of tea and that and talked it over, you’d find where the difficulties are…And that second or third visit to see would make all the difference. If you went back a week, a fortnight, later and had it out, it would be more efficient, financially as well.’ (Telecare user, Mrs K, aged 80)*

Participants also emphasised that building relationships through ongoing informal and open-ended interactions with clients would provide the service with information they needed to support and monitor the user effectively. In particular, building a relationship between the client and monitoring staff (whether face-to-face or someone at the end of the telephone) was important, because frequent and informal contact provided insight into aspects of their life that would have a bearing on the use or appropriateness of the technology (for example, illness, life events, anxieties).

*‘We had a patient recently that it was just too much for her [to use telehealth] at the moment because she’s trying to get a carer for her mother, so we put her on a break for a couple of weeks. She had all the intentions to do it, but not at that time, it wasn’t, you know, it was the last thing that she needed to do.’ (Telehealth Centre Operator)*

This subtle, granular information about the detail of technology users’ lives was felt to be difficult to capture through formal assessments or data integration. For this reason, participants concluded that services should make efforts to maintain frequent contact and exploit opportunities for interaction with users and their care network (for example, by making the most of interactions relating to ‘false alarms’, test calls and maintenance).

Technology developers acknowledged the importance of formal and informal social support networks, but they assumed these networks were usually in place to implement and support the technology effectively. However, when they considered the problems faced among ATHENE case participants, they raised a number of suggestions for greater social affordance within the design, so that assistive technologies could better facilitate the social connectivity and cohesion required to support the user alongside the technology.

*‘It’s not our job as a technology provider to create social networks for people, people can do that for themselves. We have to be aware that we don’t get in the way of those social networks…And maybe the answer isn’t to make it [the technology] as simple as possible. Maybe the answer is to make it as socially adaptive as possible. So maybe a good Telehealth system would be one that relies very strongly on* [*social networks*].’*. (Business Development Lead)*

Table 3 summarises the key dimensions of quality gleaned chiefly from the phase 3 workshops, informed and refined by findings from earlier phases.

**Table 3:** **The ‘ARCHIE’ framework of quality principles for designing, installing and supporting telehealth and telecare products and services**

**Archie: anchored, realistic, continuously co-created, human, integrated, evaluated**	
**Principle 1**	**Design and development should be ANCHORED in a shared understanding of what matters to the patient or client**
Spend time with the individual to find out what activities and functions are personally meaningful and important to them. These are often socio-culturally framed (for example,. relating to historical accounts of their lives, family or community roles, and cultural or religious practices). ‘What matters to the person’ should be shared and understood by all involved in supporting him or her. Advocacy may be needed to represent the client and ensure their needs and goals remain central.	
**Principle 2**	**The technology solution and care package should be REALISTIC about the natural history of illness and the (often progressive) impairments it may bring**
The idea that assistive technologies can cure degenerative disease or fully compensate for its effects is a modernist myth. With few exceptions, multi-morbidity steadily and inexorably compromises key aspects of functioning. Non-specific impairments (for example, chronic tiredness, loss of motivation, dulling of cognitive capacity) may interfere with a person’s ability and motivation to use a technology that has been designed to alleviate specific physical, mental or emotional impairments. Effective solutions take both the materiality and affordances (of technology) and the capability (of the user) into account.	
**Principle 3**	**Solutions should be CONTINUOUSLY CO-CREATED along with users and carers, using practical reasoning and common sense**
Personalisation of solutions should be seen as a continual process that never ends, rather than as part of a standardised, one-off assessment. Formal and informal care networks require capacity to track and review the solution while in use, recognising that further customisation and innovation are likely. Creativity is needed to deal with diverse and abnormal situations, including ‘outside the box’ thinking and practical reasoning, rather than sticking rigidly to standard protocols and procedures.	
**Principle 4**	**HUMAN elements (personal relationships, social networks) will make or break a telehealth or telecare solution**
Frequent inter-personal interactions with users and their carers (as informal as possible) will build their familiarity with the service and promote trust, a sense of being cared for and confidence to take the initiative if problems arise. Such interactions will also develop providers’ knowledge about key contextual factors that may have a bearing on delivery of effective and dependable support. Technology needs to be aligned with both formal and informal social support that can bridge the design-reality gap in ways that are sometimes very subtle. It is important to consider the available human resources within the intended user’s care network, and how members of this network might connect with the technology and service to support use and customisation.	
**Principle 5**	**The service must be INTEGRATED by maximising mutual awareness, co-ordination and mobilisation of knowledge and expertise**
Everyone involved (both lay and professional) must be clear about the patient’s or client’s changing needs and capabilities and about the technical and social supports in place. They must also have an ongoing sense of what the other collaborators are doing to provide a context for their own activity towards the common goal of supporting the person to achieve what matters to them. To that end, it is crucial to mobilise the different knowledge and expertise within the network – both formal (shared, for example, through systematic entry and exchange of data on records) and informal (shared, for example, through storytelling, inter-disciplinary case-based discussion and informal interactions).	
**Principle 6**	**EVALUATION and monitoring is essential to inform system learning**
Few telehealth and telecare programmes to date have maximised the potential to learn and improve. Technology designers and services need to monitor use and experience of technology solutions, workarounds developed for them and the repurposing of the technology and service, to inform ongoing innovation and improvements for both individual clients and the wider system.	

## Discussion

### Summary of key dimensions of quality

This paper has described qualitative research on people with a range of assisted living needs as well as the perspective of technology designers and service providers. Ethnographic studies of the user experience were particularly central in informing co-design workshops. These workshops were technology-agnostic – that is, their purpose was not to inform a specification for a particular technology but to distil more abstract design principles for both technologies and (more broadly) the services in which they are installed and used.

The ARCHIE framework in Table 3 states that telehealth and telecare products and services must be *anchored* in what matters to users; *realistic* about the natural history of illness, *continuously co-created* (developing and adapting solutions in an ongoing way with those who are using them), underpinned by strong *human* relationships and embedded in social networks; *integrated* using the principles of computer-supported cooperative work (maximising mutual awareness and mobilising knowledge and expertise across the network); and *evaluated* with a view to both single-loop learning (individual case review) and double-loop learning (improvement at organisation and system level) [37].

### Comparison with other literature

As noted in the introduction, much research in the biomedical literature on assisted living has been modernist in its vision, technological in its focus and oriented to demonstrating proof of concept – that is, that the technology ‘works’ in optimal experimental conditions, ideally in a randomised controlled trial design [3,4,9]. This study, in contrast, has aligned with research from sociological and philosophical traditions, which place central emphasis on what matters to patients. Such traditions view ‘assisted living’ as an effortful accomplishment of dynamic networks of humans and technologies, which must be optimally aligned and continually adapted to deliver success [7,11-13,15,27,38-40].

Sited within this broader literature, our findings are perhaps unsurprising. In particular, they resonate strongly with previous findings by the EFORTT research team on ‘networks of accountabilty’ in telecare [12] and with Jeanette Pols’ rich ethnographic study ‘Care at a distance’ [15]. The ethical framework for telecare proposed by the EFORTT researchers consists of seven main questions: to what extent have users been consulted and involved in the design of a telecare device?; what problems can telecare help with (and, implicitly, what can it not help with)?; who is connected to the telecare system?; how might the device change the home?; who (patient, carer and so on) will be the active user of the device?; is the effort needed to deliver the service worthwhile in this case?; and what would happen if the person’s condition deteriorated? [12].

### Strengths and limitations of this study

The main strength of this study is our use of multiple qualitative methods, combined in sequence, to build up a particularly rich picture of the challenges of designing, installing and using assistive technologies in a UK setting, giving particular emphasis to the voice of the person with assisted living needs. These methods have allowed us to capture detailed ideas and proposals for improving these processes at the level of the individual patient or client – and at organisational and sector level. Another advantage of our study is the interdisciplinary nature of the research team, which included a medical doctor (TG) and an occupational therapist (PS) as well as a psychologist (JW), computer scientist (RP) and sociologists (MR and SH). Our diverse backgrounds enabled us to combine a strong clinical focus with a robust theoretical approach to consider how people and technologies come together (or not) to meet the clinical and existential needs of a person with multi-morbidity.

The main limitation of this study is not methodological but philosophical. We have produced recommendations that are not easy to implement because they require fundamental changes in how different stakeholder groups operate and interface with one another. Not only have we not come up with a specification for a technology that will ‘fix’ the challenges of telehealth and telecare provision; we have demonstrated that no such technological fix can ever be developed. The solutions we propose, based on our findings and supported by previous findings from the EFORTT study in particular, are orders of magnitude more difficult to deliver, since they demand far-reaching changes in the organisation and delivery of services, the way health and care organisations purchase technologies, the way staff from these organisations work together on the ground, and the level of ongoing commitment by all players that will be needed to maintain an assisted living solution once it has been developed. A reviewer of an earlier draft of this paper commented that some of the problems evident in our data relating to the introduction and use of assistive technologies (and, by implication, the support of people with multiple co-morbidity more generally) stem not from the technologies themselves or their ‘implementation’ but from ‘*wider patterns of organisation and delivery of personal services, and the neo-liberal attack on the allocation of resources to them*’. We agree with this comment, though it is beyond the scope of this paper to address it in detail.

## Conclusions

Technologies hold huge potential for supporting high-quality care and independent living in people with multi-morbidity. But solutions, if they are to be scalable and sustainable, must be socio-technical (that is, technologies must be developed alongside the networked social relations that make them ‘work’) and pragmatically customised to meet people’s unique and changing medical, personal, social and cultural needs.

For the ARCHIE principles to be realised, three key shifts are required across the sector – none of which will be easy or cheap. First, both technology designers and assistive technology services need to shift their focus from developing, installing and monitoring a particular technology to a more dynamic focus on performance (supporting technologies-in-use). Second, those who commission telehealth and telecare services need to shift from standardised care packages (the one-size-fits-all ‘home care contract’) to personalised solutions (that is, they should require providers to adapt products and services to the patient’s needs and preferences). Thirdly, industry (perhaps supported by relevant incentives by government) must drive a shift in the *design model* from ‘walled garden’ branded solutions (packages that are designed to interface only with a particular manufacturer’s products) to components that are designed to be combined creatively by people making their own *ad hoc* solutions to one-off challenges, and which must, therefore, be inter-operable across multiple devices and platforms. Technological advances are important, but they must be underpinned by a robustly user-centred approach to technology design and service delivery by industry and service providers.

What we mean by ‘a robustly user-centred approach’ goes beyond existing practices where users are invited to take part in requirements gathering and evaluation processes. In relation to the former, the ‘turn to the social’ in information and communication technology design, first proposed over 20 years ago [41], emphasised the importance of understanding the context of use and led to the adoption of ethnographic methods of the kind we have used in this study. What our studies reveal, however, is that prevailing assumptions about the influence of context – both material and social – on how people use technology are inadequate. Context is not a stable feature of the setting into which a technology is to be deployed that can be defined in advance for a specific use or user; rather, context and use are dynamic and co-constitutive [42]. Industry and service providers must implement design and delivery processes where users (patients, their informal and formal carers) are able to negotiate and evolve – to co-produce – both the technologies and the services supported in and through them. We offer the six principles of the ARCHIE framework to industry and service providers as a means to help orientate them to what a robustly user-centred approach should deliver.

## Box 1: Key messages

- Telehealth and telecare technologies are not, and never will be, ‘plug and play’ or ‘one size fits all’.
- Many people who are fitted with such technologies never use them because of insufficient adaptation to the person’s physical and cognitive capabilities and/or to the personal, family, socio-cultural and organisational context(s) in which they can be made to ‘work’ (or not).
- Telehealth and telecare should be thought of as an ongoing, personalised service, supported by technology and requiring a network of lay and professional carers, not as a technology package that is installed on a one-off visit.
- We introduce the ARCHIE standards: provision of telehealth and telecare should be **A**nchored in a shared understanding of what matters to the user; **R**ealistic about the natural history of illness; **C**o-creative, evolving and adapting solutions with users; **H**uman, supported through interpersonal relationships and social networks; **I**ntegrated, through attention to mutual awareness and knowledge sharing; **E**valuated, to drive system learning.
